# Phase 1 study of selinexor plus carfilzomib and dexamethasone for the treatment of relapsed/refractory multiple myeloma

**DOI:** 10.1111/bjh.15969

**Published:** 2019-05-24

**Authors:** Andrzej J. Jakubowiak, Jagoda K. Jasielec, Cara A. Rosenbaum, Craig E. Cole, Ajai Chari, Joseph Mikhael, Jennifer Nam, Amanda McIver, Erica Severson, Leonor A. Stephens, Kathryn Tinari, Shaun Rosebeck, Todd M. Zimmerman, Tyler Hycner, Agata Turowski, Theodore Karrison, Jeffrey A. Zonder

**Affiliations:** ^1^ University of Chicago Medical Center Chicago IL USA; ^2^ Weill Cornell Medical College New York NY USA; ^3^ Department of Medicine Division of Hematology/Oncology University of Michigan School of Medicine Ann Arbor MI USA; ^4^ Tisch Cancer Institute/Multiple Myeloma Program Mount Sinai School of Medicine New York NY USA; ^5^ Mayo Clinic, Phoenix, AZ, and International Myeloma Foundation Los Angeles CA USA; ^6^ Barbara Ann Karmanos Cancer Institute Wayne State University Detroit MI USA; ^7^ Present address: Translational Genomics Research Institute City of Hope Cancer Center Phoenix AZ USA

**Keywords:** relapsed/refractory multiple myeloma, selinexor, carfilzomib, dexamethasone

## Abstract

Selinexor, an oral Selective Inhibitor of Nuclear Export, targets Exportin 1 (XPO1, also termed CRM1). Non‐clinical studies support combining selinexor with proteasome inhibitors (PIs) and corticosteroids to overcome resistance in relapsed/refractory multiple myeloma (RRMM). We conducted a phase I dose‐escalation trial of twice‐weekly selinexor in combination with carfilzomib and dexamethasone (SKd) to determine maximum tolerated dose in patients with RRMM (*N* = 21), with an expansion cohort to assess activity in carfilzomib‐refractory disease and identify a recommended phase II dose (RP2D). During dose escalation, there was one dose‐limiting toxicity (cardiac failure). The RP2D of twice‐weekly SKd was selinexor 60 mg, carfilzomib 20/27 mg/m^2^ and dexamethasone 20 mg. The most common grade 3/4 treatment‐emergent adverse events included thrombocytopenia (71%), anaemia (33%), lymphopenia (33%), neutropenia (33%) and infections (24%). Rates of ≥minimal response, ≥partial response and very good partial response were 71%, 48% and 14%, respectively; similar response outcomes were observed for dual‐class refractory (PI and immunomodulatory drug)/quad‐exposed (carfilzomib, bortezomib, lenalidomide and pomalidomide) patients (*n* = 17), and patients refractory to carfilzomib in last line of therapy (*n* = 13). Median progression‐free survival was 3·7 months, and overall survival was 22·4 months in the overall population. SKd was tolerable and re‐established disease control in RRMM patients, including carfilzomib‐refractory patients.

Registered at ClinicalTrials.gov (NCT02199665)

The development of immunomodulatory drugs (IMiDs; e.g. lenalidomide, pomalidomide) and proteasome inhibitors (PIs; e.g. carfilzomib, bortezomib) as standards of care for patients with multiple myeloma (MM) has resulted in significant improvements in survival (Brenner *et al*, 2009; Kumar *et al*, 2008; Thumallapally *et al*, 2016). However, nearly all patients require multiple lines of therapy as they relapse or develop disease refractory to treatment. First‐line and subsequent therapies usually involve IMiDs and PIs in doublet or triplet combinations with corticosteroids and other systemic therapies (Kumar *et al*, 2018). As use of these combinations have become new standards of care, the treatment challenges in the relapsed/refractory (RR) setting have evolved, with increasing numbers of patients being quad‐refractory to bortezomib, lenalidomide, pomalidomide and carfilzomib or penta‐refractory to these four drugs and the anti‐CD38 monoclonal antibody daratumumab. There is a need to develop agents with novel mechanisms of action to overcome treatment resistance (Chim *et al*, 2018; Sonneveld & Broijl, 2016).

Selinexor is an oral Selective Inhibitor of Nuclear Export that targets Exportin 1 (XPO1, also termed CRM1), the only known nuclear export protein for tumour suppressor proteins (TSPs) and cell‐cycle regulators (e.g. p53, FOXO, IκB, p21, p27), as well as eukaryotic translation initiation factor 4E (eIF4E)‐bound oncoprotein mRNAs (Conforti *et al*, 2015; Culjkovic‐Kraljacic *et al*, 2012; Das *et al*, 2015; Gravina *et al*, 2014). Overexpression of XPO1 is essential for MM cell survival (Schmidt *et al*, 2013; Tiedemann *et al*, 2012). XPO1 mediates the functional inactivation of cell‐cycle regulators and TSPs and promotes the export and translation of mRNA for key oncoproteins, including c‐MYC, BCL‐2 and Cyclin D (Culjkovic‐Kraljacic *et al*, 2012; Gandhi *et al*, 2018; Nguyen *et al*, 2012). Inhibition of XPO1 with selinexor restores nuclear localization of TSPs and cell‐cycle regulators (Nair *et al*, 2017; Tai *et al*, 2014). Selinexor elevates levels of the inhibitor of kappa B (IκBα), which forms complexes with and inhibits transcription factor nuclear factor (NF)κB, disrupting a range of signalling pathways, including inflammation, oncogenesis and cell survival. In myeloma cells, selinexor treatment has been shown to induce apoptosis, reduce levels of proto‐oncoproteins and impair osteoclastogenesis (Das *et al*, 2015; Schmidt *et al*, 2013; Tai *et al*, 2014).

Preclinical studies have provided a rationale for combining selinexor with PIs (Kashyap *et al*, 2016; Nair *et al*, 2017; Rosebeck *et al*, 2016; Turner *et al*, 2016). The addition of selinexor to a PI has a synergistic effect on cell death of myeloma cell lines and primary plasma cells derived from patients with RRMM, and the combination demonstrated greater antimyeloma activity in a murine xenograft model than either agent alone (Kashyap *et al*, 2016; Rosebeck *et al*, 2016; Turner *et al*, 2016). Selinexor‐PI combinations were associated with inhibition of BCL2 expression, increased cleavage and inactivation of AKT, activation of caspase‐10 and other caspases, and increased levels of IκBα and IκBα‐NFκB complexes, leading to neutralization of NF‐κB (Kashyap *et al*, 2016; Rosebeck *et al*, 2016; Turner *et al*, 2016). NF‐κB activation has been shown to be a mechanism of PI resistance (Lü & Wang, 2013; Markovina *et al*, 2008).

The clinical activity of selinexor as a single agent and as part of combination regimens has been demonstrated in heavily pre‐treated patients with RRMM (Chen *et al*, 2018; Vogl *et al*, 2018). Single‐agent selinexor was associated with modest activity in a phase I study with an objective response rate (ORR) of 4%, which improved to 50% when selinexor was combined with dexamethasone (Sd) at the twice‐weekly recommended phase 2 dose (RP2D) (Chen *et al*, 2018). In the subsequent phase II STORM study, the Sd combination generated ORRs of 21% for patients with quad‐refractory MM and 20% in penta‐refractory patients (Vogl *et al*, 2018). The addition of selinexor to bortezomib and dexamethasone (SVd) in the phase I/II STOMP study generated ORRs of 43% in a cohort with PI‐refractory RRMM (Bahlis *et al*, 2018).

Carfilzomib is approved for use in combination with dexamethasone for patients with RRMM (Berenson *et al*, 2014; Dimopoulos *et al*, 2016a; Siegel *et al*, 2012). Preclinical studies have demonstrated synergistic activity between selinexor and carfilzomib (Kashyap *et al*, 2016; Rosebeck *et al*, 2016; Turner *et al*, 2016), and clinical studies further support carfilzomib as a potential therapeutic partner in RRMM. Carfilzomib was active in patients with MM previously treated with or refractory to bortezomib (Berenson *et al*, 2016, 2014), and the combination of carfilzomib and dexamethasone (Kd) demonstrated improved progression‐free survival (PFS) and overall survival (OS) compared with the combination of bortezomib and dexamethasone (Vd) in patients with RRMM (Dimopoulos *et al*, 2017, 2016a). Here we describe a phase 1 multicentre, open‐label, investigator‐initiated study to determine the maximum tolerated dose (MTD) and the RP2D of twice‐weekly selinexor in combination with Kd in patients with RRMM, as well as safety, tolerability and preliminary efficacy.

## Methods

### Study design

This is a multicentre, open‐label, phase I study (ClinicalTrials.gov, NCT02199665). The primary objectives were to determine the MTD for the combination of twice‐weekly selinexor with Kd (SKd) in patients with RRMM, employing a 3 + 3 dose escalation design, followed by an expansion cohort to support a RP2D. Secondary objectives were to determine safety, tolerability and efficacy.

Patients aged ≥18 years with progressive RRMM were enrolled at five Multiple Myeloma Research Consortium sites in North America. Patients were eligible provided they had been previously treated with at least two anti‐myeloma therapies, including a PI and an IMiD, had an absolute neutrophil count ≥1·0 × 10^9^/l, a haemoglobin concentration ≥80 g/l, a platelet count ≥50 × 10^9^/l and adequate hepatic (total bilirubin ≤2 times the upper limit of normal and alanine aminotransferase ≤2·5 times the upper limit of normal) and renal function (creatinine clearance ≥30 ml/min) within 14 days of treatment initiation. Patients were excluded if they had received prior selinexor or any other anticancer therapy within 2 weeks of treatment initiation. Concurrent anticancer therapy other than steroids was not allowed. Other exclusion criteria included unstable angina or myocardial infarction within 4 months of treatment initiation, New York Heart Association Class III/IV congestive heart failure, left ventricular ejection fraction <40%, history of severe coronary artery disease, severe uncontrolled ventricular arrhythmias or uncontrolled hypertension or uncontrolled diabetes within 14 days of treatment initiation. Patients with plasma cell leukaemia, Waldenström macroglobulinaemia, POEMS (polyneuropathy, organomegaly, endocrinopathy, monoclonal protein, skin changes) syndrome or amyloidosis were also excluded.

The study was conducted in accordance with US Food and Drug Administration and International Conference on Harmonisation Guidelines for Good Clinical Practice, the Declaration of Helsinki, Health Canada, and any applicable local health authority, institutional review board or ethics committee requirements. All patients provided written informed consent.

### Schedule and dosing

**Figure 1:**
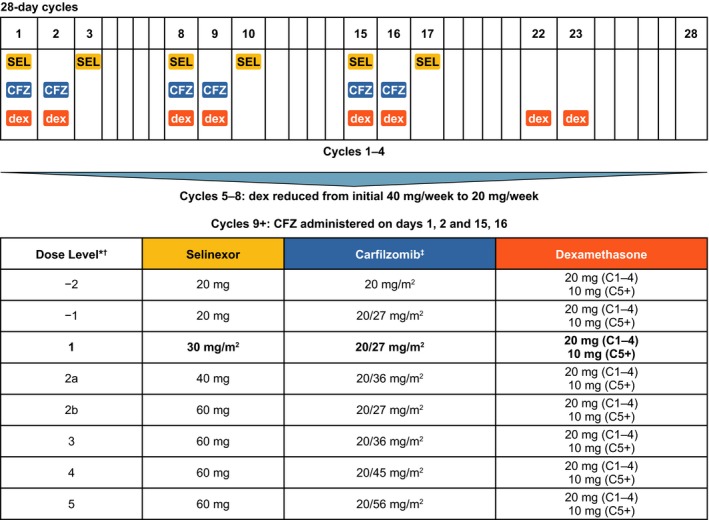
Treatment schema. *Once the Maximum Tolerated Dose was established, an expansion cohort of 6–12 patients was enrolled at that dose limited to carfilzomib‐refractory patients. ^†^Dose Level 2a and 2b were enrolled simultaneously, alternating patients between the two dose levels. ^‡^Carfilzomib initiated at 20 mg/m^2^ on Days 1–2 of Cycle 1 at all dose levels and then at the assigned dose level for the remainder of treatment. C, cycle; CFZ, carfilzomib; dex, dexamethasone; SEL, selinexor.

For the dose‐escalation phase, three patients were assigned to each cohort, beginning at Dose Level 1 (Fig 1). Selinexor, carfilzomib and dexamethasone were all administered twice weekly, and both selinexor and carfilzomib doses were escalated. Beginning at Dose Level 1, if none of the first three patients enrolled into the cohort experienced a dose‐limiting toxicity (DLT), then dose escalation proceeded to the next cohort. If any one of the three patients experienced a DLT, three more patients were added to the cohort at the same dose. If there were no additional DLTs, dose escalation proceeded to the next cohort. If two or more DLTs were observed among the initial three or expanded six patients, the dose level was considered to exceed the MTD. Because there were delays in patient accrual, the study protocol was amended to ensure that eligible patients could enrol at the time of their availability—expansion to six patients per cohort was allowed if, at a given dose level, three patients were enrolled and no DLTs were observed but all three had not completed their first cycle of treatment. Patients who did not receive all scheduled doses (unrelated to drug toxicity) during Cycle 1 were replaced for DLT evaluation per the study protocol. Given that our patients were heavily pre‐treated with advanced disease, we anticipated that disease‐related sequalae might prevent patients from completing the dose‐escalation phase without dose interruption.

Once the MTD or maximum recommend dose was determined, that dose cohort was to be expanded to a total of 12 carfilzomib‐refractory patients as defined by the International Myeloma Working Group (IMWG) criteria (Rajkumar *et al*, 2011). If the overall DLT rate for this cohort was <30%, this dose would be declared the RP2D.

All patients received herpes zoster virus prophylaxis (e.g. valacyclovir) and prophylactic anti‐emetic therapy with megesterol acetate (160–400 mg daily) and a 5‐hydroxytryptamine‐3 (5‐HT_3_) antagonist.

### Assessments

Safety and tolerability were evaluated by means of drug‐related DLTs in the dose‐escalation cohorts, treatment‐emergent adverse events (AEs), physical examinations and laboratory tests. DLTs were prespecified haematological and non‐haematological toxicities that were considered treatment‐related and occurred during Cycle 1. Haematological DLTs were: febrile neutropenia; grade 4 neutropenia lasting >7 days; grade 4 thrombocytopenia lasting >7 days despite dose delay; and grade 3/4 thrombocytopenia associated with bleeding. Non‐haematological DLTs were: grade ≥2 neuropathy and any grade ≥3 toxicity (lasting for ≥3 days for gastrointestinal events and >7 days for fatigue or anorexia) despite maximal supportive care except for electrolyte abnormalities, hair loss and elevation of alanine aminotransferase or aspartate aminotransferase. Dose modifications were allowed, but any treatment toxicities that required a dose reduction during Cycle 1, or any toxicities that delayed initiation of Cycle 2 by >7 days were also considered DLTs.

Treatment‐emergent AEs were graded according to the National Cancer Institute‐Common Terminology Criteria for Adverse Events v4.0 (National Cancer Institute 2010). Patients that discontinued treatment underwent a final assessment at 28 days after the last dose of a study drug. Patients were followed for survival for up to 2 years after the end of treatment.

Efficacy measures included response according to the IMWG criteria (Durie *et al*, 2006; Rajkumar *et al*, 2011)—progressive disease (PD), stable disease (SD), minimal response (MR), partial response (PR), very good partial response (VGPR), complete response (CR) and stringent complete response (sCR). Response assessments were completed at Cycle 2 Day 1 and Day 1 of subsequent cycles. All response criteria required confirmation with two consecutive assessments.

### Statistical analysis

Continuous and categorical variables were summarized with descriptive statistics. ORR (≥PR) and a clinical benefit rate (CBR; ≥MR) were estimated, and 90% confidence intervals (CIs) were generated for the RP2D cohort. If the data deviated strongly from normality as judged by boxplots and normal probability plots, non‐parametric, Wilcoxon signed‐rank tests were performed in place of *t*‐tests. Given the small sample size, multiplicity adjustments were not made to the alpha levels; these analyses were considered exploratory and hypothesis‐generating only. Time‐to‐event endpoints were assessed by the Kaplan–Meier method using GraphPad Prism 7.03 software (La Jolla, CA, USA).

## Results

Twenty‐one patients with RRMM were enrolled between July 2014 and September 2016. The data cut‐off for this analysis was 1 September 2017. Patients were aged between 55 and 74 years, 43% were ≥65 years of age (Table 1). The median time since the initial MM diagnosis was 4·5 years. Twelve (57%) patients were identified as high risk per IMWG criteria, including 5 (24%) with del (17p). Patients received a median of 4 (range, 2–10) prior lines of therapy. Previous treatments included carfilzomib (95%) and pomalidomide (81%) (Table 2). All patients (100%) were refractory to last line of therapy, including 13 (62%) who were refractory to carfilzomib (4 to carfilzomib/dexamethasone, 9 to carfilzomib/pomalidomide/dexamethasone). Seventeen (81%) patients were dual‐class refractory (refractory to a PI and an IMiD) and quad‐exposed (bortezomib, carfilzomib, lenalidomide and pomalidomide). Baseline characteristics and prior therapies by dose level are presented in Tables SI and SII.

**Table 1:** Patient characteristics

Characteristic	*N* = 21
Age	
Median years (range)	64 (55–74)
≥65 years, *n* (%)	9 (43)
Sex, *n* (%)	
Male	11 (52)
Female	10 (48)
Time since diagnosis, median (range), years	4·5 (1·6–11·7)
ECOG performance status, *n* (%)	
0	13 (62)
1	8 (38)
ISS stage, *n* (%)	
I	2 (10)
II	7 (33)
III	4 (19)
Unknown	8 (38)
Cytogenetic risk per IMWG, *n* (%)	
High†	12 (57)
Deletion 17p	5 (24)
Standard	9 (43)

ECOG, Eastern Cooperative Oncology Group; IMWG, International Myeloma Working Group; ISS, International Staging System.

† Defined per IMWG: t(4;14), del(17p), t(14;16), t(14;20), non‐hyperdiploidy and gain(1q).

**Table 2:** Prior therapies

Prior therapy	*N* = 21
Prior lines of therapy, median (range)	4 (2–10)
Prior PIs, *n* (%)	21 (100)
Carfilzomib	20 (95)
Bortezomib	20 (95)
Prior IMiDs, *n* (%)	21 (100)
Lenalidomide	20 (95)
Pomalidomide	17 (81)
Thalidomide	4 (19)
Other prior therapies, *n* (%)	20 (95)
Autologous stem‐cell transplantation	20 (95)
Panobinostat	2 (10)
Daratumumab	1 (5)
Refractory to prior therapy, *n* (%)	21 (100)
Carfilzomib	20 (95)
Bortezomib	11 (52)
Pomalidomide	17 (81)
Lenalidomide	14 (67)
Dual‐class refractory/quad‐exposed†	17 (81)
Triple‐class refractory/penta‐exposed‡	1 (5)
Refractory in last line of therapy, *n* (%)	21 (100)
Carfilzomib	13 (62)
Pomalidomide	11 (52)
Carfilzomib and pomalidomide	9 (43)

IMiD, immunomodulatory drug; PI, proteasome inhibitor.

† Refractory to a PI and an IMiD; exposed to bortezomib, lenalidomide, carfilzomib and pomalidomide.

‡ Refractory to a PI, an IMiD and an anti‐CD38 antibody; exposed to bortezomib, lenalidomide, carfilzomib, pomalidomide and daratumumab.

Of the 21 patients enrolled, 18 were evaluable for DLTs (received one full course of treatment or stopped treatment due to a DLT). All 21 patients were included in overall toxicity, survival and response assessments. One patient with a history of congestive heart failure (CHF) was retrospectively determined to be ineligible due to pre‐existing amyloidosis which was unknown at study entry. This patient enrolled in the dose‐escalation phase of the study and was included in safety and efficacy assessments.

At data cut‐off, the median duration of treatment was 4 cycles (range, 1–14 cycles), with 20 (95%) patients completing at least 1 cycle and 11 (52%) completing at least 4 cycles. Twenty patients discontinued the study due to disease progression (17 [81%]), patient or physician's choice (1 [5%]) and treatment toxicities (2 [10%]).

### Determination of the MTD

During the course of Cycle 1, there were no DLTs at Dose Level 1 (30 mg/m^2^ selinexor; 20/27 mg/m^2^ carfilzomib; 20 mg dexamethasone). Two patients in the Dose Level 1 cohort did not receive all scheduled treatment doses (i.e. dose modifications unrelated to toxicity) and were replaced per the study protocol, resulting in a total of five patients in this cohort. There were no DLTs in three patients enrolled at Dose Level 2a (40 mg selinexor; 20/36 mg/m^2^ carfilzomib; 20 mg dexamethasone). A DLT was experienced by one of the first three patients enrolled at Dose Level 2b (60 mg selinexor; 20/27 mg/m^2^ carfilzomib; 20 mg dexamethasone); the patient with a history of CHF who retrospectively was found to have cardiac amyloidosis experienced cardiac failure during Cycle 1. Therefore, three additional patients were assigned to the cohort. One patient did not receive all scheduled doses (unrelated to toxicity) and was replaced, resulting in a total of seven patients for Dose Level 2b, with no additional DLTs during the dose escalation stage.

Further dose escalation was not pursued based on AE rates, tolerability and anti‐myeloma activity. Assessment by dose level showed rapid disease control at all dose levels but no notable trend in the rate or depth of response to support further escalation, while rates of some AEs increased after Cycle 1, as did dose reduction. In the safety population, the rate of grade 3/4 anaemia was 5% during Cycle 1 but increased to 33% after Cycle 1. In the dose escalation cohorts, dose reductions were required by 80% (4/5) of patients enrolled at Dose Level 1, 100% (3/3) at Dose Level 2A, and 29% (2/7) at Dose Level 2b (Table SIII). Based on these findings and previous experience with selinexor (Bahlis *et al*, 2018; Chen *et al*, 2018; Vogl *et al*, 2018), Dose Level 2b was selected as the maximum recommended dose and selected for expansion. Six additional patients were enrolled into the Dose Level 2b cohort for a total of 13 patients, of whom 12 were carfilzomib‐refractory. Among these 6 patients, there were 2 DLTs during Cycle 1 (grade 3 diarrhoea and grade 3 decrease in platelet count), yielding a DLT rate of 25% for the 12 patients who completed 1 cycle of Dose Level 2b (below the predefined limit of 30%). Dose Level 2b was selected as the RP2D.

### Safety

In the 21 enrolled patients, the most commonly observed treatment‐emergent adverse events (TEAEs) of any grade were thrombocytopenia (81%), fatigue (81%) and anaemia (71%). The most frequently observed grade 3/4 haematological TEAEs were thrombocytopenia (71%), anaemia (33%), lymphopenia (33%) and neutropenia (33%), and the most common grade 3/4 non‐haematological TEAEs included infections (24%), fatigue (14%), diarrhoea (10%), eye disorders (10%), musculoskeletal disorders (10%) and elevated liver enzymes (10%). Decreased appetite, weight loss and anorexia occurred in 5%, 5% and 29% of patients, respectively, and all were grade 1/2 in severity. Table 3 summarises TEAEs overall and by dose level. Serious AEs included upper‐respiratory tract infection (*n* = 3), urinary tract infection (*n* = 2), mastoid osteomyelitis (*n* = 2), upper gastrointestinal (GI) bleeding (*n* = 1; deemed unrelated to treatment with a platelet count of 167 × 10^9^/l at the time of the event), syncope (*n* = 1), deep vein thrombosis and pulmonary embolism (*n* = 1), pain related to PD (*n* = 1) and CHF with ejection fraction decrease (*n* = 1) considered related to carfilzomib treatment in the patient with a history of CHF and who retrospectively did not meet eligibility criteria due to amyloidosis.

**Table 3:** Treatment‐emergent adverse events

	Overall *N* = 21		Dose Level 1 30 mg/m^2^ SEL; 20/27 mg/m^2^ CFZ; 20 mg DEX *n* = 5		Dose Level 2a 40 mg SEL; 20/36 mg/m^2^ CFZ; 20 mg DEX *n* = 3		Dose Level 2b† 60 mg SEL; 20/27 mg/m^2^ CFZ; 20 mg DEX *n* = 13	
	All Grades	Grades 3/4	All Grades	Grades 3/4	All Grades	Grades 3/4	All Grades	Grades 3/4
	*n* (%)	*n* (%)	*n* (%)	*n* (%)	*n* (%)	*n* (%)	*n* (%)	*n* (%)
Haematological								
Thrombocytopenia	17 (81)	15 (71)	4 (80)	4 (80)	3 (100)	3 (100)	10 (77)	8 (62)
Anaemia	15 (71)	7 (33)	4 (80)	2 (40)	2 (67)	0	9 (69)	5 (38)
Lymphopenia	11 (52)	7 (33)	3 (60)	2 (40)	1 (33)	1 (33)	7 (54)	4 (31)
Neutropenia	8 (38)	7 (33)	3 (60)	3 (60)	2 (67)	2 (67)	3 (23)	2 (15)
Non‐haematological								
Fatigue	17 (81)	3 (14)	4 (80)	3 (60)	3 (100)	0	10 (77)	0
Dyspnoea	11 (52)	1 (5)	3 (60)	0	3 (100)	1 (33)	5 (38)	0
Nausea	11 (52)	0	4 (80)	0	2 (67)	0	5 (38)	0
Diarrhoea	10 (48)	2 (10)	2 (40)	0	2 (67)	0	6 (46)	2 (15)
Musculoskeletal disorders	8 (38)	2 (10)	4 (80)	2 (40)	1 (33)	0	3 (23)	0
Eye disorders	7 (33)	2 (10)	3 (60)	1 (20)	2 (67)	0	2 (15)	1 (8)
Infection	6 (29)	5 (24)	1 (20)	1 (20)	1 (33)	1 (33)	4 (31)	3 (23)
Anorexia	6 (29)	0	3 (60)	0	0	0	3 (23)	0
Elevated liver and pancreatic enzymes	8 (38)	2 (10)	4 (80)	1 (20)	1 (33)	0	3 (23)	1 (8)
Vomiting	5 (24)	0	2 (40)	0	0	0	3 (23)	0
Oedema	4 (19)	1 (5)	2 (40)	1 (20)	1 (33)	0	1 (8)	0
Hyponatraemia	2 (10)	1 (5)	1 (20)	1 (20)	0	0	1 (8)	0
Confusion	2 (10)	1 (5)	1 (20)	1 (20)	0	0	1 (8)	0
Decreased appetite	1 (5)	0	0	0	1 (33)	0	0	0
Weight loss	1 (5)	0	0	0	1 (33)	0	0	0
Psychosis	1 (5)	1 (5)	1 (20)	1 (20)	0	0	0	0
Syncope	1 (5)	1 (5)	0	0	0	0	1 (8)	1 (8)

CFZ, carfilzomib; DEX, dexamethasone; SEL, selinexor.

† Recommended phase 2 dose.

Dose modifications included new cycle delays for 11 (52%) patients, dose interruptions for 17 (80%) and dose reductions for 13 (62%). Dose modification was needed for selinexor in 15 (71%) patients, for carfilzomib in 11 (52%) and for dexamethasone in 7 (33%). Treatment was discontinued in two patients due to toxicity, which included a patient with a urinary tract infection and the patient with the pre‐existing amyloidosis who experienced CHF (the latter was considered treatment‐related). Two patients experienced progressive myeloma while treatment was on hold because of AEs (pneumonia and cytopenias, respectively).

### Response and treatment outcomes

Most patients achieved disease control after 1 cycle (CBR of 67% and ORR of 38%). For best response during the course of treatment, the CBR was 71%, ORR was 48% and the VGPR rate was 14% (Table 4). There were no CRs. The patient with pre‐existing amyloidosis who experienced a DLT discontinued SKd prior to response evaluation; this was considered a non‐response. For patients receiving the RP2D (*n* = 13), CBR was 62% (90% CI: 0·36–0·83), ORR was 38% (90% CI: 0·17–0·65) and the rate of VGPR was 15% (90% CI: 0·03–0·41); two additional patients achieved SD. For dual‐refractory/quad‐exposed patients (*n* = 17), the CBR was 76%, ORR was 53% and the VGPR rate was 18%, and for patients who were refractory to carfilzomib in last line of therapy (*n* = 13), the corresponding values were 77%, 62% and 15%. The one patient who was tri‐refractory/penta‐exposed achieved VGPR.

**Table 4:** Response rates

	Overall	Dose Level 1 30 mg/m^2^ SEL; 20/27 mg/m^2^ CFZ; 20 mg DEX	Dose Level 2a 40 mg SEL; 20/36 mg/m^2^ CFZ; 20 mg DEX	Dose Level 2b† 60 mg SEL; 20/27 mg/m^2^ CFZ; 20 mg DEX
Best response, *n* (%)	*N* = 21	*n* = 5	*n* = 3	*n* = 13
Complete response	0	0	0	0
Very good partial response	3 (14)	1 (20)	0	2 (15)
Partial response	7 (33)	2 (40)	2 (67)	3 (23)
Minimal response	5 (24)	1 (20)	1 (33)	3 (23)
Stable disease	2 (10)	0	0	2 (15)
Progressive disease	3 (14)	1 (20)	0	2 (15)
Non‐response‡	1 (5)	0	0	1 (8)
ORR, *n* (%)	10 (48)	3 (60)	2 (67)	5 (38)
CBR, *n* (%)	15 (71)	4 (80)	3 (100)	8 (62)
Carfilzomib refractory in last line of therapy§, *n* (%)	*n* = 13	*n* = 4	*n* = 2	*n* = 7
Very good partial response	2 (15)	1 (25)	0	1 (14)
ORR	8 (62)	3 (75)	2 (100)	3 (43)
CBR	10 (77)	3 (75)	2 (100)	5 (71)
Dual class refractory/quad exposed¶, *n* (%)	*n* = 17	*n* = 4	*n* = 3	*n* = 10
Very good partial response	3 (18)	1 (25)	0	2 (20)
ORR	9 (53)	3 (75)	2 (67)	4 (40)
CBR	13 (76)	4 (100)	3 (100)	6 (60)

CBR, clinical benefit rate (≥minimal response); CFZ, carfilzomib; DEX, dexamethasone; DLT, dose‐limiting toxicity; IMiD, immunomodulatory drug; ORR, objective response rate (≥partial response); PI, proteasome inhibitor; RP2D, recommended phase 2 dose; SEL, selinexor; VGPR, very good partial response.

† Recommended phase 2 dose.

‡ Patient was not evaluable due to a DLT that resulted in treatment discontinuation prior to response evaluation.

§ Refractory to carfilzomib at ≥20 mg/m^2^ on twice‐weekly schedule (i.e. on Days 1, 2, 8, 9, 15 and 16).

¶ Refractory to PI and IMiD/exposed to bortezomib, lenalidomide, carfilzomib and pomalidomide.

**Figure 2:**
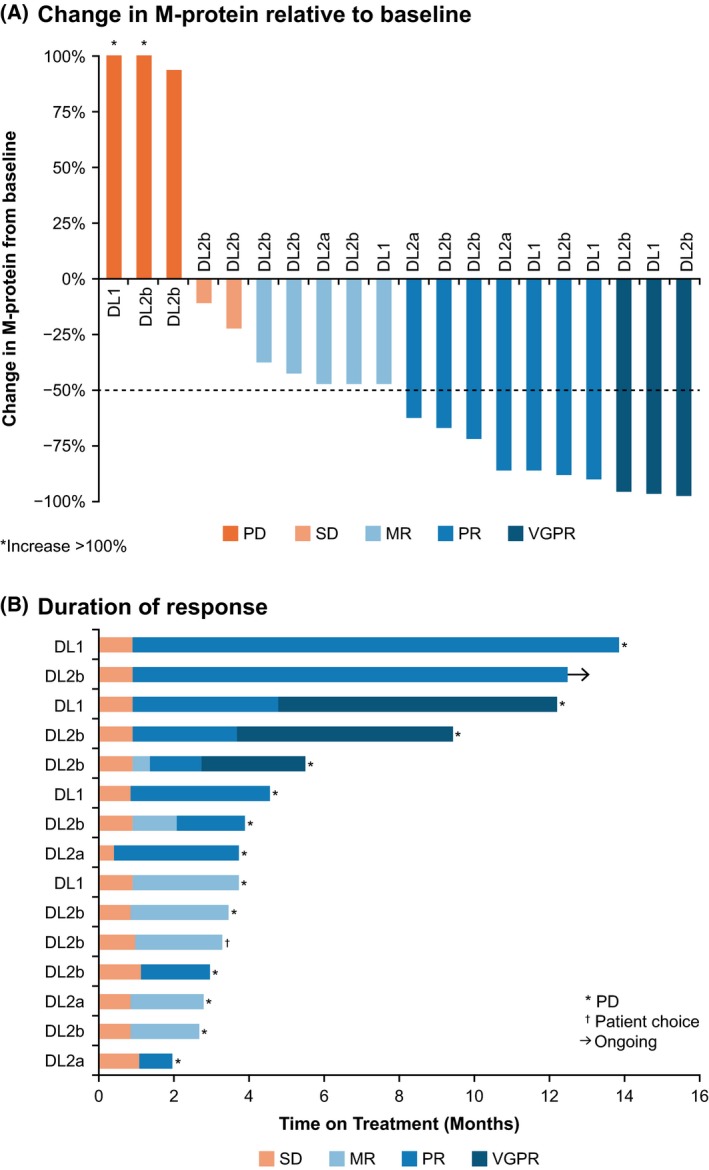
Depth and duration of response in evaluable patients (*n* = 20). (A) Change in M‐protein relative to baseline. *Increase >100%. One patient with dose‐limiting toxicity that resulted in treatment discontinuation prior to response evaluation was not included. (B) Duration of response. Three patients had PD at first response assessment and were not included; two patients remained in SD and were not included; one patient with dose‐limiting toxicity that resulted in treatment discontinuation prior to response evaluation was not included. DL, dose level; MR, minimal response; PD, progressive disease; PR, partial response; SD, stable disease; VGPR, very good partial response.

Durability and depth of responses are presented in Fig. 2. The median duration of response for patients who achieved ≥MR and ≥PR were 2·9 and 3·4 months, respectively, for all response‐evaluable patients, 3·1 and 3·0 months for the RP2D cohort, 2·8 and 3·3 months for the carfilzomib‐refractory cohort and 3·1 and 3·0 months for the high‐risk cohort.

**Figure 3:**
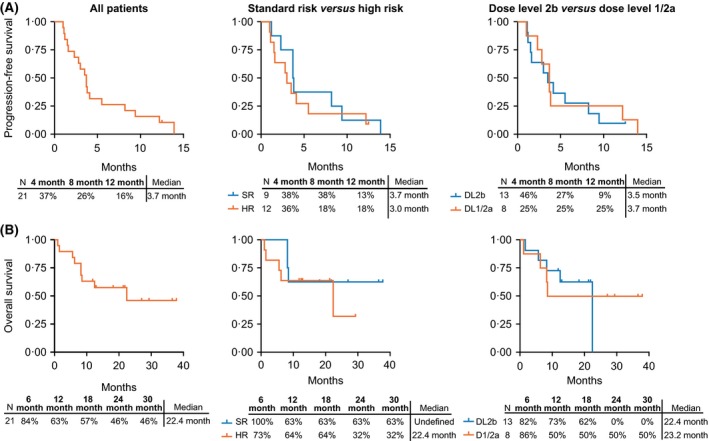
Kaplan–Meier estimated progression‐free survival (A) and overall survival (B) in all patients (*N* = 21) and by International Myeloma Working Group ‐risk status and dose level. DL, dose level; HR, high risk; mo, months; SR, standard risk.

Median PFS and OS were 3·7 and 22·4 months, respectively, for all enrolled patients (Fig. 3), 3·7 and 22·4 months for the carfilzomib‐refractory cohort and 3·0 and 22·4 months for the high‐risk cohort. Median PFS and OS were 3·5 and 22·4 months for the R2PD cohort and 3·7 and 23·2 months for the patients enrolled at Dose Level 1 or 2a.

## Discussion

This study demonstrated the safety and tolerability of twice‐weekly SKd. Overall, the combination demonstrated manageable tolerability. Most patients required dose modifications, but only two patients discontinued due to treatment‐related AEs. All patients received prophylactic megestrol acetate and a 5‐HT_3_ antagonist treatment to mitigate GI AEs. Additional supportive measures, including growth factors and transfusions, could be initiated by the investigator at any time during treatment. The most common grade 3/4 AEs were haematological in nature. GI and constitutional AEs were common but were generally grade 1/2. Although carfilzomib is associated with cardiac AEs (Chen *et al*, 2017b; Waxman *et al*, 2018), only one patient (with a history of CHF and underlying cardiac amyloidosis) experienced a cardiac AE considered related to carfilzomib that led to treatment discontinuation. Based on the overall tolerability and the anti‐myeloma activity of the regimen, Dose Level 2b (twice‐weekly selinexor 60 mg with a standard twice‐weekly dosing of carfilzomib at 20/27 mg/m^2^, and twice‐weekly dexamethasone at 20 mg) was selected for dose expansion, without determination of the MTD. Based on the high rate of dose reductions during our study and results from prior selinexor studies (Bahlis *et al*, 2018; Chen *et al*, 2018; Vogl *et al*, 2018), we concluded that further dose escalation would probably not be feasible. Dose Level 2b was clinically active with a DLT rate <30% and selected as the RP2D.

The safety results with SKd were generally consistent with safety outcomes from previous studies of selinexor in RRMM, although the rates and severity of non‐haematological AEs, particularly GI events, were lower than anticipated (Bahlis *et al*, 2018; Chen *et al*, 2018; Vogl *et al*, 2018). In the phase II STORM study (*N* = 79 RRMM), the most common grade 3/4 treatment‐related AEs associated with twice‐weekly Sd (80 mg selinexor/20 mg dexamethasone) was thrombocytopenia (59%) (Vogl *et al*, 2018), an established toxicity of selinexor because of its inhibition of megakaryocyte maturation (Machlus *et al*, 2017). Other grade 3/4 treatment‐related AEs included anaemia (28%), neutropenia (23%) and lymphopenia (11%). Treatment‐related non‐haematological AEs (any grade) included nausea (73%), fatigue (63%), decreased appetite (49%), anorexia (49%), vomiting (44%), diarrhoea (43%) and weight loss (33%); these were generally grade 1/2 in severity (Vogl *et al*, 2018).

In the phase I/II STOMP study (*N* = 42 RRMM), selinexor was administered twice‐weekly at 60 or 80 mg or once‐weekly at 80 or 100 mg in combination with Vd during the dose‐escalation stage; this was followed by a dose expansion with the RP2D (Bahlis *et al*, 2018). The investigators selected 100 mg once‐weekly dose of selinexor as the RP2D, and nearly all patients (39/42) received bortezomib 1·3 mg/m^2^ weekly at treatment initiation rather than at the standard twice‐weekly dose schedule. The results suggested that the rates for some treatment‐related haematological and GI AEs improved with once‐weekly selinexor. The rate of grade 3/4 thrombocytopenia decreased from 69% during the dose‐escalation stage (60 or 80 mg selinexor twice‐weekly/once‐weekly) to 31% with the RP2D, anaemia decreased from 25% to 4%, and grade 3 diarrhoea decreased from 13% to 4% (Bahlis *et al*, 2018).

The clinical activity of SKd is promising and compares favourably with activity in studies of selinexor alone and selinexor with dexamethasone (Sd) (Chen *et al*, 2018; Vogl *et al*, 2018). However, we also recognize the limitations of cross‐study comparisons and differences between study populations. The current study enrolled heavily pre‐treated patients, all of which were refractory to their last line of therapy: 95% were refractory to carfilzomib, 62% were refractory to carfilzomib in the last line of therapy and 57% had high‐risk cytogenetics. In a phase I study of patients with RRMM (*n* = 81) or Waldenström macroglobulinaemia (*n* = 3), selinexor (3–60 mg/m^2^ or fixed dose of 40 or 60 mg) showed modest activity as a single agent with an ORR of 4% (*n* = 57) and a CBR of 21%, which improved to 22% and 33%, respectively, when twice‐weekly selinexor (45 or 65 mg/m^2^) was combined with 20 mg of dexamethasone (*n* = 27). At the 45 mg/m^2^ selinexor dose, the ORR for the combination was 50% (Chen *et al*, 2018). In the STORM study, twice‐weekly Sd (80/20 mg) resulted in an ORR of 21% in patients with quad‐ or penta‐refractory RRMM (Vogl *et al*, 2018). Response rates with SKd also appear consistent with those of SVd from the STOMP study. ORR with SVd was 63% overall and 43% for PI‐refractory patients (*n* = 21) compared with 84% for PI‐nonrefractory patients (*n* = 19) (Bahlis *et al*, 2018).

The clinical activity of SKd also can be related to the results from historical studies in RRMM (Berdeja *et al*, 2015; Berenson *et al*, 2016, 2014; Dimopoulos *et al*, 2016b; Papadopoulos *et al*, 2015; Richardson *et al*, 2013, 2014; San Miguel *et al*, 2013; Shah *et al*, 2015; Siegel *et al*, 2012). Many of these studies enrolled patients who had been previously treated with bortezomib or lenalidomide with dexamethasone to assess the efficacy and safety of carfilzomib and pomalidomide regimens. Response rates have ranged from 32% to 77% for pomalidomide or carfilzomib in combination with dexamethasone (Berenson *et al*, 2016; Dimopoulos *et al*, 2016b; Papadopoulos *et al*, 2015; San Miguel *et al*, 2013) and 50% for the triplet of carfilzomib, pomalidomide and dexamethasone (Shah *et al*, 2015).

While response rates and time to response in our study are quite promising given the refractory status of the patient population, duration of response and PFS were shorter than anticipated in view of other selinexor studies. However, response rates, duration of response and PFS in these other selinexor studies were generally less robust in patients who were refractory to one of the drugs used in combination (Bahlis *et al*, 2018; Chen *et al*, 2017a; Gasparetto *et al*, 2017; White *et al*, 2017). Median PFS was 9·0 months for all evaluable patients in the STOMP study but 6·1 months for PI‐refractory patients, compared with 17·8 months for PI‐nonrefractory patients (Bahlis *et al*, 2018). Further, OS in our study appeared prolonged at a median of 22·4 months when considering PFS results. It is possible that SKd selected for less aggressive MM clones at the time of progression, allowing for a durable response with subsequent therapy (e.g., daratumumab), or clones that were sensitive to subsequent treatment.

Observations from this study, particularly consistent and rapid disease control (67% CBR in the first cycle), indicate that SKd could be a ‘bridge’ to subsequent therapy, allowing patients to at least transiently overcome resistance, restore disease control and prepare for subsequent therapy. Further studies are needed to better understand these observations and to determine the mechanism for loss of disease control and to evaluate whether a different dosing or schedule with SKd can improve durability of response. Based on observations from the STOMP trial (Bahlis *et al*, 2018), we are currently evaluating once‐weekly SKd in carfilzomib‐refractory, carfilzomib/PI‐naïve and non‐refractory patient populations. Other studies in RRMM are assessing selinexor in combination with liposomal doxorubicin (NCT02186834) and ixazomib and dexamethasone (NCT02831686), and additional cohorts from the STOMP trial have demonstrated the tolerability and activity of twice‐ or once‐weekly selinexor with dexamethasone in combination with lenalidomide (SRd), pomalidomide (SPd) or daratumumab (SDd) (Chen *et al*, 2017a; Gasparetto *et al*, 2017; White *et al*, 2017). The ongoing phase III BOSTON study (NCT02831686) is evaluating SVd in patients with RRMM who are PI‐relapsed/‐naïve.

With more patients receiving first‐ and second‐line combinations of PIs, IMiDs and monoclonal antibodies, it is becoming more challenging to effectively treat patients in the RRMM setting (Nooka *et al*, 2015). The addition of selinexor to Kd demonstrated manageable safety and tolerability and promising efficacy in a heavily pre‐treated population of patients with RRMM. Significant clinical activity was observed in dual‐refractory/quad‐exposed patients, and in patients who were carfilzomib‐resistant in their last line of therapy. Further studies are needed to understand the high rate of response but relatively short response duration, and to identify patients for whom SKd might serve as an effective ‘bridge’ regimen to subsequent therapies or as a line of therapy that provides durable disease control.

## Conflicts of interest

A.J.J. has received research funding from Bristol‐Myers Squibb, Amgen and Karyopharm and has received personal fees (advisory board, consultancy, speaking and honoraria) from Bristol‐Myers Squibb, Celgene, Millennium, Novartis, Amgen, SkylineDx, Karyopharm and Sanofi‐Aventis. C.A.R. received research funding from Novartis, Millennium, GlaxoSmithKline and Bristol‐Meyers Squibb; received honoraria, travel accommodations and expenses from Bristol‐Myers Squibb and Celgene. C.E.C. received travel, accommodations and expenses from Amgen. A.C. holds a consultancy/advisory role for Amgen, Array Biopharma, Bristol‐Myers Squibb, Celgene, Janssen Oncology, Millennium and Novartis; received research funding from Acetylon Pharmaceuticals (Inst), Array BioPharma, Biotest (Inst), Bristol‐Myers Squibb (Inst), Celgene, Janssen, Millennium, Novartis, Onyx and Pharmacyclics LLC, an AbbVie Company; and received travel accommodations and expenses from Amgen, Bristol‐Myers Squibb, Celgene, Janssen Oncology, Novartis and Takeda. J.M. received research funding from AbbVie, Celgene and Sanofi. A.M. and L.A.S. are employed with and have ownership interests in TG Therapeutics. T.M.Z. is employed with AbbVie. J.A.Z. holds a consultancy/advisory role for Amgen, Array BioPharma, Bristol‐Myers Squibb, Celgene, Janssen, Prothena, Seattle Genetics and Takeda; and received research funding for Celgene (Inst). The remaining authors declare no competing financial interests.

## Authorship contributions

Designed study: A.J.J., J.K.J., K.T. T.K. Collected data: A.J.J., C.A.R., C.E.C., A.C., J.M., J.N., A.M., E.S., L.A.S., K.T., S.R., T.M.Z., T.H., A.T., J.A.Z. Performed statistical analyses: A.J.J., T.H., T.K. Confirmed accuracy of data: A.J.J., A.C., T.H., A.T., T.K., J.A.Z. Drafted manuscript: A.J.J., J.A.Z. All authors reviewed the draft manuscript and approved the final manuscript for submission.

## Supporting information


**Table SI.** Patient characteristics by dose level.
**Table SII.** Prior therapies by dose level.
**Table SIII.** Dose modifications by dose level.
