# Supplementary material for: Phase 1 study of selinexor plus carfilzomib and dexamethasone for the treatment of relapsed/refractory multiple myeloma
Source: Br J Haematol. 2019 May 24;186(4):549–60. doi: 10.1111/bjh.15969 (PMC6772147; doi:10.1111/bjh.15969)
Supplement: Supplementary file 1 — Table SI. Patient characteristics by dose level. Table SII. Prior therapies by dose level. Table SIII. Dose modifications by dose level. [file BJH-186-549-s001.docx]

# Supplemental Information

# Phase 1 study of selinexor plus carfilzomib and dexamethasone for the treatment of relapsed/refractory multiple myeloma

Andrzej J. Jakubowiak, Jagoda K. Jasielec, Cara A. Rosenbaum, Craig E. Cole, Ajai Chari, Joseph Mikhael, Jennifer Nam, Amanda McIver, Erica Severson, Leonor A. Stephens, Kathryn Tinari, Shaun Rosebeck, Todd M. Zimmerman, Tyler Hycner, Agata Turowski, Theodore Karrison, Jeffrey A. Zonder

**Table S1: Patient Characteristics by Dose Level**

| **Characteristic** | **Overall**  N=21 | **Dose Level 1**  30 mg/m^2^ selinexor; 20/27 mg/m^2^ CFZ; 20 mg dex  n=5 | **Dose Level 2a**  40 mg selinexor; 20/36 mg/m^2^ CFZ; 20 mg dex  n=3 | **Dose Level 2b^a^**  60 mg selinexor; 20/27 mg/m^2^ CFZ; 20 mg dex  n=13 |
| --- | --- | --- | --- | --- |
| Age |  |  |  |  |
| Median years (range) | 64 (55–74) | 64 (59–73) | 67 (55–68) | 62 (55–74) |
| ≥65 years, n (%) | 9 (43) | 2 (40) | 2 (67) | 5 (38) |
| Sex, n (%) |  |  |  |  |
| Male | 11 (52) | 2 (40) | 2 (67) | 7 (54) |
| Female | 10 (48) | 3 (60) | 1 (33) | 6 (46) |
| Time since diagnosis, median (range), years | 4.5 (1.6–11.7) | 3.6 (2.7–8.6) | 2.8 (1.6–5.9) | 5.2 (2.0–11.7) |
| ECOG performance status, n (%) |  |  |  |  |
| 0 | 13 (62) | 4 (80) | 2 (67) | 7 (54) |
| 1 | 8 (38) | 1 (20) | 1 (33) | 6 (46) |
| ISS stage, n (%) |  |  |  |  |
| I | 2 (10) | 1 (20) | 0 | 1 (8) |
| II | 7 (33) | 2 (40) | 1 (33) | 4 (31) |
| III | 4 (19) | 1 (20) | 1 (33) | 2 (15) |
| Unknown | 8 (38) | 1 (20) | 1 (33) | 6 (46) |
| Cytogenetic risk per IMWG, n (%) |  |  |  |  |
| High^b^ | 12 (57) | 2 (40) | 1 (33) | 9 (69) |
| Deletion 17p | 5 (24) | 0 | 1 (33) | 4 (31) |
| Standard | 9 (43) | 3 (60) | 2 (67) | 4 (31) |

^a^Recommended phase 2 dose

^b^Defined per IMWG: t(4;14), del(17p), t(14;16), t(14;20), non-hyperdiploidy and gain(1q).

ECOG, Eastern Cooperative Oncology Group; IMWG, International Myeloma Working Group; ISS, International Staging System.

**Table S2: Prior Therapies by Dose Level**

| **Prior therapy** | **Overall**  N=21 | **Dose Level** **1**  30 mg/m^2^ selinexor; 20/27 mg/m^2^ CFZ; 20 mg dex  n=5 | **Dose Level 2a**  40 mg selinexor; 20/36 mg/m^2^ CFZ; 20 mg dex  n=3 | **Dose Level 2b^a^**  60 mg selinexor; 20/27 mg/m^2^ CFZ; 20 mg dex  n=13 |
| --- | --- | --- | --- | --- |
| Prior lines of therapy, median (range) | 4 (2–10) | 5 (2–5) | 2 (2–5) | 4 (2–10) |
| Prior PIs, n (%) | 21 (100) | 5 (100) | 3 (100) | 13 (100) |
| Carfilzomib | 20 (95) | 5 (100) | 3 (100) | 12 (92) |
| Bortezomib | 20 (95) | 4 (80) | 3 (100) | 13 (100) |
| Prior IMiDs, n (%) | 21 (100) | 5 (100) | 3 (100) | 13 (100) |
| Lenalidomide | 20 (95) | 5 (100) | 2 (67) | 13 (100) |
| Pomalidomide | 17 (81) | 4 (80) | 3 (100) | 10 (77) |
| Thalidomide | 4 (19) | 1 (20) | 0 | 3 (23) |
| Other prior therapies, n (%) | 20 (95) | 5 (100) | 3 (100) | 12 (92) |
| Autologous stem-cell transplantation | 20 (95) | 4 (80) | 3 (100) | 13 (100) |
| Panobinostat | 2 (10) | 1 (20) | 0 | 1 (8) |
| Daratumumab | 1 (5) | 0 | 0 | 1 (8) |
| Refractory to prior therapy, n (%) | 21 (100) | 5 (100) | 3 (100) | 13 (100) |
| Carfilzomib | 20 (95) | 5 (100) | 3 (100) | 12 (92) |
| Bortezomib | 11 (52) | 3 (60) | 1 (33) | 7 (54) |
| Pomalidomide | 17 (81) | 4 (80) | 3 (100) | 10 (77) |
| Lenalidomide | 14 (67) | 4 (80) | 0 | 10 (77) |
| Dual-class refractory/quad-exposed^b^ | 17 (81) | 4 (80) | 2 (67) | 11 (85) |
| Triple-class refractory/penta-exposed^c^ | 1 (5) | 0 | 0 | 1 (8) |
| Refractory in last line of therapy, n (%) | 21 (100) | 5 (100) | 3 (100) | 13 (100) |
| Carfilzomib | 13 (62) | 4 (80) | 2 (67) | 7 (54) |
| Pomalidomide | 11 (52) | 3 (60) | 3 (100) | 5 (38) |
| Carfilzomib and pomalidomide | 9 (43) | 2 (40) | 2 (67) | 5 (38) |

^a^Recommended phase 2 dose

^b^Refractory to a PI and an IMiD; exposed to bortezomib, lenalidomide, carfilzomib and pomalidomide.

^c^Refractory to a PI, an IMiD and an anti-CD38 antibody; exposed to bortezomib, lenalidomide, carfilzomib, pomalidomide and daratumumab.

**Table S3: Dose modifications by dose level**

|  | **Overall**  N=21 | **Dose Level** 1  30 mg/m^2^ selinexor; 20/27 mg/m^2^ CFZ; 20 mg dex  n=5 | **Dose Level** **2a**  40 mg selinexor; 20/36 mg/m^2^ CFZ; 20 mg dex  n=3 | **Dose Level** **2b^a^**  60 mg selinexor; 20/27 mg/m^2^ CFZ; 20 mg dex  n=13 |
| --- | --- | --- | --- | --- |
| New cycle delays, n (%) | 11 (52) | 3 (60) | 2 (67) | 6 (46) |
| Dose holds, n (%) | 17 (81) | 4 (80) | 3 (100) | 10 (77) |
| Dose reduction, n (%) | 13 (62) | 4 (80) | 3 (100) | 6 (46)^b^ |

^a^Recommended phase 2 dose

^b^Dose reductions required by 2/7 patients enrolled during the dose-escalation stage and 4/6 patients enrolled during the expansion stage
